# Exposure Assessment for Some Pesticides through Rice Consumption in Iran Using a Multiresidue Analysis by GC-MS

**Published:** 2018

**Authors:** Maryam Amirahmadi, Hassan Yazdanpanah, Farzad Kobarfard, Shahram Shoeibi, Morteza Pirali-Hamedani, Hossein Rastegar

**Affiliations:** a *Food* *and* *Drug* *Laboratories* *Research* *Center, Ministry* *of* *Health* *and* *Medical* *Education, Tehran, Iran*; b *.* *Food* *and* *Drug* *Control* *Laboratories, Food* *and* *Drug* *Administration, Ministry* *of* *Health* *and* *Medical* *Education, Tehran, Iran*; c *.* *Food* *Safety* *Research* *Center, Shahid* *Beheshti* *University* *of* *Medical* *Sciences, Tehran, Iran. *; d *Department* *of* *Toxicology* *and* *Pharmacology, School* *of* *Pharmacy, Shahid* *Beheshti* *University* *of* *Medical* *Sciences, Tehran, Iran. *; e *Department* *of* *Medicinal* *Chemistry, School* *of* *Pharmacy, Shahid* *Beheshti* *University* *of* *Medical* *Sciences, Tehran, Iran*; f *.* ^*f*^ *Phytochemistry* *Research* *Center, Shahid* *Beheshti* *University* *of* *Medical* *Sciences, Tehran, Iran.*; g *Department* *of* *Medicinal* *Chemistry, School* *of* *Pharmacy, Tehran* *University* *of* *Medical* *Sciences, Tehran, Iran.*

**Keywords:** Pesticide residues, Exposure assessment, Spiked calibration curve, GC/MS-SIM, Rice, Iran

## Abstract

In communities which consume rice as main food, importance of risk assessment for contaminants is always taken into consideration by health authorities. The present study is an attempt for monitoring of 56 pesticides from different chemical groups in rice samples collected from local markets in Tehran and estimation of daily intake of interested pesticides through this monitoring. A valid method based on spiked calibration curves and QuEChERS sample preparation was developed for determination of pesticides residue in rice by GC/MS. The analytical results of the proposed method were in good agreement with the proficiency test (FAPAS 0969). One-hundred-thirty-five rice samples were analyzed and 11 pesticide residues were found in 10.4% of the samples. Of which 5.2% were contaminated with unregulated pesticides. None of the samples, which were contaminated with regulated pesticides, had contamination higher than maximum residue limit. The mean estimated dose (ED) was calculated with respect of mean of contamination and mean daily consumption of rice. ED of the found pesticides is much lower than the related ADIs.

## Introduction

Cereal crops constitute more than 60% of the total worldwide agricultural production (1). More than 90% of the world’s rice is cultured and consumed in Asia (2). Rice is a major food crop for more than 60% of the world’s population. The consumption of rice in Iran is 110 g per capita/day (3).

 Rice is a pesticide-intensive crop; pesticides are applied either directly to the soil prior to planting and flooding of rice fields, or a few weeks after flooding to control noxious weeds and pests (4).

Exposure assessment is necessary to reach correct Maximum Residue Levels (MRLs) for consumer health assurance. Intake of pesticides residue in food is obtained by multiplying the residue level in the food by the amount of that food consumed. The total dietary intake of the pesticide residue is then calculated by summing the intakes of all foods containing the residue. The estimated dietary intake resulting from application of a pesticide and other sources should be less than its established Acceptable daily intake (ADI) (5). Risk assessment for pesticides residue in traditional way is based on applying of individual pesticides. However, human beings are exposed daily to multiple pesticides and the risk of their exposure which acts in a similar way can be characterized by cumulative risk assessment (6). 

The increasing public concern about pesticide contamination of food and the environment in recent years has increased the demand for broader and stricter pesticide monitoring. Therefore, it is necessary to develop rapid, reliable, and effective analytical methods for the simultaneous determination of the residues of pesticides in order to obtain accurate information about the types and quantity of the pesticides used (7). Importance of pesticides residue monitoring in rice could be proved by focusing of a few recently published articles (8-12).

In this study we investigate the levels of 56 pesticides residue in rice with a rapid multi-residue method of analysis based on a QuEChERS extraction procedure using spiked calibration. The selected pesticides included GC-amenable pesticides, those for which MRL is issued by Iranian National Standards Organization (INSO) (13) and Codex alimentarius commission and the most frequently reported pesticides in rice by FDA during 1996-2006 survey (14).

The developed method was used for simultaneous determination of the selected pesticides in 135 domestic and imported rice samples collected from Tehran retail market. Three major components of the process of dietary pesticide risk assessment are estimation of pesticide residue levels, estimation of food consumption patterns, and characterization of risk based on a comparison of exposure estimates with toxicological criteria. Dietary pesticide risk assessment and estimation of pesticides daily intake have been the focus of a few recently published articles (15-20).

Estimated daily intake (EDI) is a parameter of calculating the amount of contact with pesticides in each day. There are four main approaches for collecting food consumption data which are Household-based methods, Population-based methods, Individual-based methods, and combined methods. This Estimated Dose (ED) of detected pesticides in adults was determined using residue concentration data obtained from survey, combined with the consumption of rice for adult (60 kg body weight) in Iran (21). To evaluate the health risk of estimated dietary pesticide exposure, it was compared with ADIs set by JMPR (22). Exceeding the ADI may indicate potential harm and require further evaluation.

## Experimental

*Chemicals*

All pesticides standards were purchased from Dr. Ehrenstorfer Co. (Augsburg, Germany). All organic solvents, intended for extraction, were at least LC grade and purchased from Merck (Darmstadt, Germany). Bulk quantities of NaCl were obtained from Merck (Darmstadt, Germany). Anhydrous MgSO_4_ was obtained from SIGMA-Aldrich CO. (Japan). The MgSO_4_ was baked for 5 h at 500 ºC in a furnace to remove phthalates and residual water. Primary secondary amine (PSA) was purchased from Supelco (Bellefonte, USA).

*GC–SQ/MS*

An Agilent Technologies 6890N Network GC System chromatograph (Wilmington, USA) with a SQ detector and equipped with an Agilent 7683B autosampler (Agilent technologies, USA) was used. A HP-5 capillary column (30 m × 0.25 mm I.D., 1 μm film thickness) was used for separation.

*Calibration standards*

Individual stock standard solutions (1 mg/mL) were prepared in ethyl acetate and stored in the dark at −20 °C. They were kept for 1 h at ambient temperature prior to their use. A mixed stock standard solution of pesticides was prepared in ethyl acetate at 15 μg/mL with respect to each pesticide. Spiked calibration curves at 7 levels of 10, 25, 50, 100, 250, 500 and/or 1000 ng/g triplicate were prepared by addition of 10 μL, 25 μL, 50 μL, 100 μL, 250 μL, 500 μL, and/or 1000 μL of mixed standard stock solution, respectively, to 15 g portions of blank rice samples in each case. 

A stock solution of triphenylmethane (TPM) in ethyl acetate at concentration of 1 mg/mL was used as internal standard and an aliquot of 10 μL of TPM solution in ethyl acetate was added to the spiked rice sample. The samples so obtained were treated as described in sample preparation section. 

*Sample preparation*

A domestic sample purchased from under controlled filed and analyzed 5 times for ensuring blank samples. An aliquot of 10 μL of internal standard solution (1000 mg/L) was added to 15 g of milled (Romer mill, USA) blank rice sample in a 50 mL falcon tube and after being left for 1 h at ambient temperature in dark, 15 mL acetonitrile was added. The mixture was mixed at high speed with vortex mixer for 1 min. One gram of NaCl and 2 grams of activated anhydrous MgSO_4 _was added to the mixture, and mixing was continued for an additional 60 sec. The mixture was centrifuged for 5 min at 4000 rpm at -5 °C. The supernatant was transferred to a 15 mL falcon tube containing 1 g MgSO_4_ and 300 mg PSA. After shaking for 1 min and centrifugation for 5 min at 4000 rpm at -5 °C, 4 mL of supernatant was transferred to a 5 mL vial and evaporated to ca. 0.3 mL under a gentle stream of nitrogen gas. The residue was reconstituted by toluene to obtain 1 mL solution, and after shaking for 3 min, 2 μL of the solution was injected into gas chromatograph. 

*Recovery studies*

For recovery determination, spiked blank rice samples at concentration levels of 10, 25, 50, 100, 250, 500, and 1000 ng/g were prepared in triplicates and they were kept for 1 h at ambient temperature prior to their use and then treated according to the procedure described in sample preparation section. The recoveries were calculated using the calibration curves constructed using spiked samples. 

*GC-SQ–MS analysis*

The GC-SQ-MS was employed with helium as the carrier gas at a constant flow of 1 mL/min. The oven temperature started at 75 °C and remained at this temperature for 3 min increasing to 120 °C at 25 °C/min ramp rate and then increased to 300 °C at 5 °C/min ramp, holding at 300 °C for 11 min. Injection port was adjusted at 250 °C and splitless mode was used.

After acquisition of the total ion chromatogram for the mixed stock standard solutions in scan mode, peaks were identified by their retention time and mass spectra. The identification was confirmed by comparing the relative abundances for three-four ions (one quantifier and two-three qualifiers) of the experimental standards to known relative abundances of the Pest Library reference spectra. The most abundant ion that showed no evidence of chromatographic interference and had the highest signal-to-noise ratio was selected for quantification purposes. 

*Quantitation*

The concentrations of pesticides were determined by interpolation of the relative peak areas for each pesticide to internal standard peak area in the sample on the spiked calibration curve. 

*Validation with a proficiency test (FAPAS) of a rice sample*

We participated in the proficiency test organized by the Food Analysis Performance Assessment Scheme of the Central Science Laboratory York (UK) in March 2011. (FAPAS 0969) (23). Each participant received a rice test material to be analyzed for pesticide. The result reported by our laboratory for pesticides in dispatched test material with Z-score (-0.1 and zero for Malathion and pirimiphos- methyl respectively) successfully met requirements of the organization. The result supported accuracy of the improved method for quantification of pesticides.

*Application to real samples*

One-hundred-thirty-five rice samples were collected from local markets in Tehran. 

Sample size was 1 kg and during one year in each month 11-13 samples were collected from retail markets and stored in -27 °C until analysis. In order to avoid any possible thermal decomposition of pesticide residues, 200 g rice sample was mixed with 100 g dry ice and milled with Romer mill (Stylemaster Drive, USA). A 15 g portion of the sample was subjected to the process of sample preparation described in sample preparation section. 

*Estimated Dose (ED)*

The Estimated Daily Intake (EDI) as an effective item in risk assessment studies represents the total exposure from all known or suspected exposure pathways for an average person. For pesticides, Estimated Dose (ED) is related to exposure for each type food depends on the pesticide content in food and the amount of food consumed. In this study estimated dose (EDs) of the detected pesticides in adults was determined using the mean of residue concentration, combined with the amount of daily consumption of rice in Iran. ED is calculated according to the following formula (24);

ED=C × CR BW

Where,

ED = Estimated Dose is generally the number of milligrams of the contaminant that enter the body for each kilogram of body weight (mg/kg/day).

C = Mean Concentration of the interested pesticide.

CR = Contact Rate, typical units for food eaten are grams per day (g/day).

BW = Body Weight: The average body weight of an individual in kilograms (kg).

## Results

*Gas chromatographic determination*

Analysis was performed in the SIM mode based on the use of one target and two-three qualifier ions. Pesticides were identified according to their retention times and target and qualifier ions. The quantitation was based on the peak area ratio of the targets to that of internal standard. Table 1 summarizes studied pesticides with their target and qualifier ions used in SIM mode in this study.

**Figure 1:**
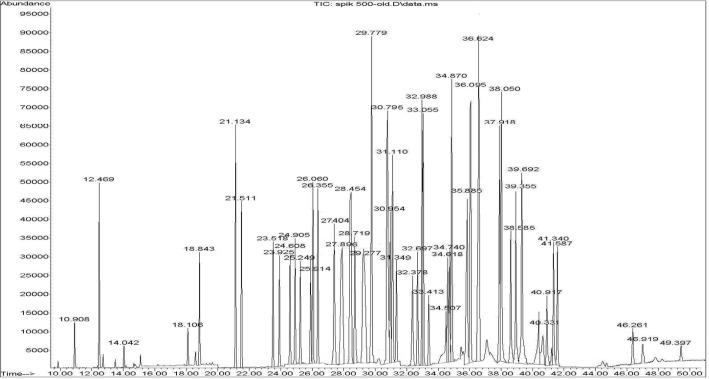
A representative chromatogram obtained for the 56 pesticides in a rice sample spiked at 500 ng/g and internal standard (Triphenyl methane, Rt = 29.77 min

Calibration curves were constructed for each compound using blank rice sample spiked at six or seven different concentration levels in triplicate. For identification of pesticides, the retention time, and three-four ions (one for quantitation and two-three for identification) were used. A GC–SQ–MS chromatogram of 56 pesticides and internal standard (TPM) analyzed in spiked rice is shown in Figure 1.

*Method validation*

*Linearity of the calibration curves*

The fifty-six pesticides showed linearity in SIM mode. Linear spiked calibration curves for all the interest pesticides in two range, 10-500 ng/g and 10-1000 ng/g were obtained with correlation factors >0.997. The Calibration data (Equation and regression coefficient) of 56 pesticides in spiked rice calibration curves is showed in Table 2.

*Limits of detection and limits of quantification*

Limits of detection (LODs) and limits of quantification (LOQs) of the proposed method were measured in spiked samples and calculated by considering a value 3 and 10 times that of background noise, respectively. The LODs and LOQs for all the pesticides were ≤10 ng/g and ≤25 ng/g, respectively, except for deltamethrin (LOD = 30 ng/g and LOQ = 90 ng/g). Table 3 shows limit of quantification (ng/g) for studied pesticides.

*Recovery*

Table 4 presents the recovery and repeatability for seven concentration levels of pesticides. The recovery of pesticides at 7 concentration levels triplicates was in the range of 96.5-104.6%. In terms of repeatability, majority of the pesticides gave RSD < 20% with n = 21 at each spiking level. The recoveries and repeatabilities are in accordance with the criteria set by SANCO Guideline (25).

*Matrix effect*

The matrix can affect the chromatographic response to the analyte. The effects depend on the nature of both matrices and the analyte. The effect is measured as an area ratio of signal in the matrix-matched standard to that in standard solution. The matrix effects were determined for the 56 pesticides at 200 ng/g concentration level. As presented in Table 5, the mean value of the matrix effect was 89.72 ± 17.7% and ranged from 51.65% to 141.5%. In this study, spike calibration curves were established for overcoming the matrix effects.

*Pesticide residues in real samples*

One-hundred-thirty-five samples were milled and analyzed according to the method described above. Fourteen (10.4%) of the 135 samples showed contamination with carbaryl, diazinon, deltamethrin, pirimiphos-methyl, piperonyl botuxide, permethrin and/or malathion (Table 6). The concentrations of diazinon, chlorpryfos, permethrin, malathion, and pirimiphos-methyl were below and for deltamethrin was above the MRLs of these pesticides in rice in Iran. No MRL is issued for the other detected pesticides in rice in Iran.

**Figure 2:**
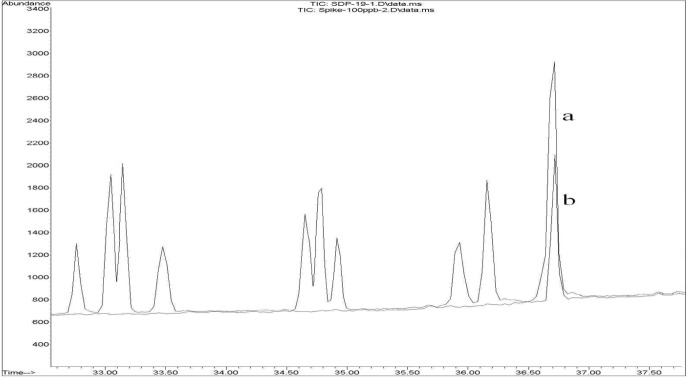
(a) An overlaid GC-MS-SIM chromatogram of a rice sample spiked at 100 ng/g of piperonyl butoxide and (b) a contaminated rice sample with piperonyl butoxide in SIM mode

Figure 2 shows (a) the overlaid chromatogram of a spiked rice sample at 100 ppb levels and (b) a contaminated rice sample in SIM mode.

*Estimated Dose (ED)*

The estimated dose (ED) of the detected pesticides in adults was determined using the mean of residue concentration, combined with the amount of daily consumption of rice in Iran. Table 7 compares the mean estimated dose of detecting pesticides with the acceptable daily intake (ADIs) established by JMPR (2006). As it appears in this table the intakes of eleven detected pesticides found in this study are much lower than the ADIs for them. Seven rice samples (5.2%) were contaminated with unregulated pesticides.

## Discussion

The use of mass spectrometry, with its information-rich content and explicit confirmation, is recommended for monitoring pesticide residues in the entire world (8-11, 26-28). Matrix-induced response enhancement was first described by Erney *et al.* in GC analysis methods (29). Since an effective elimination of the sources of the matrix induced response enhancement is not likely in practice, the analysts often try to compensate for the effect using alternative calibration methods such as matrix match calibration and standard addition methods. In the present study, we used spiked calibration curves approach to overcome the problems caused by the matrix. In this approach, calibration curves are prepared by the addition of standard solution to blank rice samples and these samples subjected to the same sample preparation procedure which is intended to be used for unknown samples. This way, the standard sample matrices will have the same composition as the unknown samples and therefore the effect of matrix is reflected in both standards and unknown samples. The calibration curve is constructed using these spiked calibration standards and it is easily used to calculate the concentration of analyte (s) in unknown sample without being concerned about the matrix effects. The recoveries and repeatabilities were in accordance with the criteria set by SANCO Guideline (25).

**Table 1:** The retention time, diagnostic ions and selected quantification ion for the target pesticides and internal standard

**No. **	**Compound**	**Diagnostic ions**	**Quantification ion**	**Retention time (min)**
1	Propoxure 1	152.1, 110.1, 209	110.1	10.907
2	Dichlorvous	220, 109, 185,145	109.0	12.468
3	Captan	151,79.1, 267,149, 107	151.0	18.097
4	Carbaryl	144, 115.1, 125.9, 116.1	144.0	18.834
5	Propoxure 2	152.1, 110.1, 209	110.1	21.128
6	Diphenyl amine	169, 168.1, 167.1	169.0	21.512
7	Alpha HCH	218.9, 182.9, 216.9, 180.9	180.9	23.511
8	Dimethoate	143, 125, 93	125.0	23.911
9	Gamma HCH	218.8, 182.9, 109	218.8	24.609
10	Beta HCH	218.9, 182.9, 109	218.9	24.898
11	Diazinon	304, 276, 179	304.0	25.245
12	Etrimfos	292.1, 277, 181.1	292.1	25.910
13	Chlortalonil	266, 264, 228.9, 267.9	266.0	26.055
14	Pirimicarb	238.2, 166.1, 138	166.1	26.373
15	Chlorpyrifos methyl	286, 125, 323, 168, 288	286.0	27.398
16	Metalaxyl	206.2, 160.1, 132.1	206.2	27.773
17	Heptachlor	336.9, 271.8, 236.9, 100	336.9	27.898
18	Alderin	263, 262.9, 264.9	262.9	27.961
19	Fentirothion	277, 260, 214,276.1	277.0	28.461
20	Pirimiphos methyl	305, 290, 276,180	305.0	28.461
21	Malathion	285, 173, 158	173.0	28.711
22	Fenthion	278, 262.9,169,153	278.0	29.212
23	Chlorpyrifos	314, 257.8, 197,199	314.0	29.337
24	Triphenyl methan	244, 165	244	29.775
25	Bioalthrin	123.1, 107.1, 91.1	123.1	30.766
26	Fipronil	420, 367, 351, 255	367.0	30.858
27	Heptachlor-exo-epoxide	352.8, 262.9, 236.8, 238.8	352.8	30.950
28	Tridimenol 1	168.1, 128, 112	168.1	31.072
29	Heptachlor-endo-epoxide	252.9, 236.9,238.9, 234.8	252.9	31.103
30	Tridimenol 2	168.1, 128, 112	168.1	31.348
31	Fenamiphos	303.2, 288, 260.1, 154	303.2	32.372
32	Alpha-Endosulfan	236.9, 264.9, 338.9	236.9	32.420
33	Hexaconazol	257.9, 233, 214, 175	214.0	32.693
34	Oxadiazon	344.1, 302, 258, 174.9	258.0	32.994
35	4,4 DDE	317.9, 246,176	246.0	33.049
36	Dieldrin	279, 262.9, 236.9	262.9	33.410
37	Iprodione	244.1, 187, 246.1, 189	187.0	34.467
38	Beta-Endosulfan	339.1, 264.9, 236.9	236.9	34.627
39	Ethion	384.1, 231, 175	231.0	34.737
40	2,4 DDT	235, 199, 165.1	235.0	34.865
41	Propiconazole 1	259, 190.9, 172.9,175	259.0	35.840
42	Edifenphos	200.9, 310, 173	310.0	35.908
43	Propiconazole 2	259, 190.9, 172.9,175	259.0	36.094
44	4,4 DDT	235, 199.1, 176.1, 237	235.0	36.099
45	Propargite	350.2, 335.2, 201.1	350.2	36.560
46	Teboconazole	249.9, 125, 296.8, 252.2	249.9	36.589
47	Piperonyl botuxide	193, 176.1, 149.1,177.1	176.1	36.624
48	Bromopropylate	340.9, 184.9, 342.9, 182.9	340.9	37.926
49	Fenpropathrin	265.1, 208, 181.1, 206.1	181.1	38.045
50	Tetradifon	355.9, 228.9, 159, 226.9	355.9	38.997
51	Phosalone	367, 182, 154, 183.9	182.0	39.301
52	Lambda cyhalothrin	208, 181.1, 227.2, 199.3	197.0	39.302
53	Permethrin 1	183.1, 127,163.1,153	183.1	41.329
54	Permethrin 2	183.1, 127,163.1,153	183.1	41.585
55	Fenvalerate 1	419.2, 225.1, 167	225.1	46.261
56	Fenvalerate 2	419.2, 225.1, 167	225.1	46.919
57	Deltamethrin	281, 252.9,255, 522	252.9	49.397

**Table 2:** Calibration data (equation and regression coefficient) for two range of 56 pesticides in spiked rice calibration curves

**Compound**	**Equation**	**Regression Coefficient**
Propoxur 1,2	*y = 0.9785x + 0.0021	0.998
	**y = 0.9743x + 0.0049	0.998
Dichlorvos	y = 0.5155x - 0.0017	0.999
	y= 0.5548x - 0.0045	0.999
Captan	y = 0.0418x - 00005	0.999
	y = 0.0421x - 00009	0.999
Carbaryl	y = 0.1617x - 00003	0.999
	y = 0.1674x - 0.0007	0.999
Diphenyl amine	y = 0.4282x + 0.0016	0.998
	y = 0.4404x + 0.0001	0.999
Beta HCH	y =0.1216x + 0.0007	0.999
	y = 0.1297x - 0.0002	0.999
Dimethoate	y = 0.2244x - 0.0012	0.999
	y = 0.2017x + 0.0015	0.997
Gamma HCH	y = 0.1206x + 0.0014	0.999
	y = 0.133x - 00008	0.998
Alpha HCH	y = 0.1356x + 0.0006	0.999
	y = 0.1485x - 0.001	0.998
Diazinon	y = 0.0995x + 0.0002	0.999
	y = 0.1032x - 0.0003	0.999
Etrimfos	y = 0.1502x + 0.0002	0.999
	y = 0.1598x - 0.001	0.999
Chlortalonil	y = 0.2652x - 0.002	0.998
	y = 0.2754x - 0.0032	0.999
Pirimicarb	y = 0.6021x + 0.0006	0.999
	y = 0.6404x - 0.004	0.999
Chlorpyrifos-methyl	y = 0.3077x - 0.0004	0.999
	y = 0.3172x - 0.0015	0.999
Metalaxyl	y = 0.2734x + 0.0022	0.999
	y = 0.2892x + 0.0003	0.999
Heptachlor	y = 0.1403x - 0.0001	0.999
Alderin	y = 0.0142x + 0.0019	0.998
	y = 0.0174x + 0.0016	0.998
Fenitrothion	y = 0.2311x - 0.0007	0.999
Pirimiphos-methyl	y = 0.155x + 0.00004	0.999
	y= 0.1656x - 0.0012	0.999
Malathion	y = 0.1895x + 0.0009	0.999
	y = 0.186x + 0.0009	0.999
Fenthion	y = 0.3424x - 0.001	0.999
	y = 0.3451x - 0.0013	0.999
Chlorpyrifos	y = 0.1192x + 0.0004	0.999
	y=0.1298x - 0.0009	0.999
Bioalthrin	y = 1.16x - 0.0023	0.999
	y=1.2203x - 0.0069	0.999
Fipronil	y = 0.2816x + 0.0004	0.999
Heptachlor-exo-epoxide	y = 0.1422x + 0.0002	0.999
	y= 0.1493x - 0.0007	0.999
Triadimenol 1,2	y = 0.2432x + 0.0009	0.997
Heptachlor-endo-epoxide	y = 0.288x - 0.0008	0.999
	y= 0.3009x - 0.0024	0.999
Fenamiphos	y = 0.2161x - 0.0006	0.999
	y= 0.237x - 0.0028	0.999
Alpha-Endosulfan	y = 0.0664x - 00008	0.999
Hexaconazol	y = 0.1717x - 0.0004	0.999
Oxadiazon	y = 0.3511x - 0.0019	0.999
	y = 0.3579x - 0.0027	0.999
4,4 DDE	y = 0.7672x - 0.0015	0.999
	y = 0.7992x - 0.0054	0.999
Dieldrin	y = 0.0907x - 0.0004	0.999
	y = 0.0912x - 0.0005	0.999
Iprodione	y = 0.0744x - 0.0005	0.999
	y = 0.1895x + 0.0009	0.999
Beta-Endosulfan	y = 0.1735x - 0.0002	0.999
2,4 DDT	y = 0.4826x - 0.0011	0.999
	y = 0.5003x - 0.0032	0.999
Ethion	y = 0.3493x - 0.0019	0.999
	y = 0.3625x - 0.0035	0.999
4,4 DDT	y = 0.3561x - 0.0015	0.999
	y = 0.4205x - 0.009	0.997
Edifenphos	y = 0.1307x - 0.0003	0.999
	y = 0.1357x - 0.0009	0.999
Propiconazole1, 2	y = 0.2099x + 0.0003	0.999
Propargite	y = 0.0658x - 0.0004	0.999
Teboconazole	y = 0.2269x - 0.0018	0.999
	y = 0.2264x - 0.0017	0.999
Piperonyl botuxide	y = 0.7256x + 0.0024	0.999
Bromopropylate	y = 0.2108x - 0.0007	0.999
Fenpropathrin	y = 0.2108x - 0.0007	0.999
Tetradifon	y = 0.211x - 00004	0.998
Phosalone	y = 0.342x + 0.0009	0.999
Lambda cyhalothrin	y = 0.4504x - 0.0003	0.999
Permethrin 1,2	y = 0.7962x + 0.0144	0.999
Fenvalerate 1,2	y = 0.0873x + 0.00005	0.999
Deltamethrin	y = 0.0118x + 0.0039	0.999

* calibration range: 10-500 ng/g.

** calibration range: 10-1000 ng/g.

**Table 3:** Limits of quantification (ng/g) for studied pesticides

**Compound**	**LOQ**	**Compound**	**LOQ**
Propoxure 1	10	Tridimenol 2	20
Dichlorvous	5	Alpha-Endosulfan	10
Captan	10	Hexaconazol	5
Carbaryl	10	Oxadiazon	25
Propoxure 2	5	4,4 DDE	5
Diphenyl amine	5	Dieldrin	10
Beta HCH	5	Iprodione	25
Dimethoate	8	Beta-Endosulfan	5
Gamma HCH	5	Ethion	20
Alpha HCH	5	2,4 DDT	5
Diazinon	10	Propiconazole 1	10
Etrimfos	10	Edifenphos	10
Chlortalonil	10	Propiconazole 2	10
Pirimicarb	5	4,4 DDT	5
Metalaxyl	5	Teboconazole	15
Heptachlor	10	Piperonyl botuxide	5
Alderin	25	Bromopropylate	10
Fentirothion	20	Fenpropathrin	8
Pirimiphos methyl	8	Tetradifon	20
Malathion	12	Phosalone	10
Fenthion	10	Lambda cyhalothrin1	15
Chlorpyrifos	10	Lambda cyhalothrin2	10
Bioalthrin	10	Permethrin 1	10
Fipronil	3	Permethrin 2	10
Heptachlor-exo-epoxide	5	Fenvalerate 1	10
Tridimenol 1	10	Fenvalerate 2	15
Heptachlor-endo-epoxide	5	Deltamethrin	90
Fenamiphos	10		

**Table 4:** Average recoveries (%) and range of relative standard deviations (%) of pesticides obtained by GC-MS analysis of rice samples at 7 spiking levels (n = 3).

**Compound**	**Average recovery (%) (n=3)**							**Total recovery (%) (n = 21)**	**Range of RSD%**
	**10 (ng/g)**	**25 (ng/g)**	**50 (ng/g)**	**100 (ng/g)**	**250 (ng/g)**	**500 (ng/g)**	**1000 (ng/g)**		
Propoxure 1, 2	115.5	94	102.7	91.7	106.8	98.6	102.1	99.3	3.2-19.7
Dichlorvous	126.9	99.4	102.8	91.9	101.1	102.8	101	99.9	5.3-11.7
Captan	118	102.5	93.3	95.4	102.2	99.7	100.1	98.9	3.1-15.0
Carbaryl	108.7	95.2	97.9	96.2	103.3	99.4	103.7	98.7	2.9-18.7
Diphenyl amine	100.3	111.8	106	104	92.9	101.5	100.5	102.8	4.6-23.4
Alpha HCH	86.5	83.4	93.7	103.3	104.4	98.8	101.8	97.6	0.1-20.3
Dimethoate	113.6	112.1	92.6	97.4	95.8	101.2	97.6	99.4	0.5-21.7
Gamma HCH	102.4	76.1	95.9	101.1	105	98.7	101.99	96.5	1.9-13.7
Beta HCH	94.2	83.8	94.7	104	102.8	99.2	101.2	97.6	4.5-20.2
Diazinon	101.4	90.6	90.2	107.5	100.4	99.7	100.7	98.7	2.1-15.4
Etrimfos	99.2	89.4	96.5	103.6	101	99.6	101.3	98.6	3.6-12.4
Chlortalonil	119.6	105.7	115.5	97.5	92.4	101.8	100.8	102.3	3.7-26.3
Pirimicarb	100.6	91.6	97.9	101.8	101.1	99.7	106.9	99.8	2.4-18.4
Chlorpyrifos methyl	114.8	104.8	109.8	101.2	92.6	101.6	100.6	101.8	2.8-25.9
Metalaxyl	72.4	90.9	102.4	98.6	96.7	100.8	101.2	98.5	3.8-15.7
Heptachlor	108.8	88.4	83.1	101.8	104.7	99.7	99.8	96.3	1.9-25.2
Alderin	74.9	117.3	102.8	93.2	99.2	105.6	98.7	102.8	4.9-22.2
Fentirothion	121.5	93.2	101.2	96.7	94.4	104.9	99.2	101.6	1.1-16.0
Pirimiphos methyl	103.9	90.5	91.2	102	103.3	99.2	101.4	98.8	2.4-15.8
Malathion	70	80.4	105.6	108.4	98.7	94.8	98.7	98.7	4.2-27.5
Fenthion	120.4	102.2	105.9	101.4	94.9	101.1	100.2	100.9	1.1-19.6
Chlorpyrifos	101.1	83.8	90.1	96.9	99.7	100.3	109.7	97.4	0.6-16.2
Bioalthrin	117.2	97.8	104	95.3	100.6	100.3	100.1	102.2	1.6-16.9
Fipronil	104.2	94.9	107.8	103.4	98.3	98.8	100.4	101.1	1.5-12.8
Heptachlor-exo-epoxide	97.5	92.6	98.5	98.1	103.4	99.3	101.1	98.6	0.8-11.4
Tridimenol	119.8	112.9	104.3	97.43	84.13	109.93	98.53	98.5	4.5-12.4
Heptachlor-endo-epoxide	115	98.4	103.1	96.8	98.5	100.4	100.9	101.9	1.3-15.0
Fenamiphos	119.8	91.1	98.3	101.3	99.9	103.1	110.3	101	0.8-11.8
Alpha-Endosulfan	117.9	75.4	102.6	96.8	101.5	100.7	99.7	99.2	5.4-26.4
Hexaconazol	115.3	106.4	102.5	95.5	99.5	100.2	102.2	103.1	2.1-22.8
Oxadiazon	122.7	112.1	100.1	97.2	97.7	100.6	100.4	104.4	3.5-15.0
4,4 DDE	118.2	94.8	100.8	100.1	98.8	100.3	100.8	101.9	1.0-7.8
Dieldrin	123.5	110.2	102.9	97.3	97.8	100.1	100.1	104.6	0.4-12.4
Iprodione	119.9	109.9	99.1	97.4	98.5	100.4	98.5	103.4	1.8-9.3
Beta-Endosulfan	107.5	102.9	88	108.2	91.3	104.5	99.4	100.3	7.6-19.2
Ethion	122.7	106.5	103.2	97.7	97.3	100.7	100.6	104.1	1.2-11.3
2,4 DDT	116.8	97.4	99.8	97.5	100.3	100	100.75	101.8	4.6-16.3
Propiconazole 1, 2	112.5	102.8	93.5	95.2	97.2	103.6	99.3	100.6	0.8-11.3
Edifenphos	120.7	101.8	103.2	96.9	98.8	100.4	100.8	103.2	2.1-11.9
4,4 DDT	110.9	85.9	99.6	99.6	100	89.2	103	98.3	1.8-13.5
Propargite	118.9	115.7	98.8	89.5	97.7	103.4	99.4	103.4	0.8-8.8
Teboconazole	120.4	116.1	105.1	91.5	98.9	100.5	99.7	104.6	1.5-19.1
Piperonyl botuxide	78.4	97.2	104.3	100.9	107.4	94.5	100.8	97.6	2.0-22.8
Bromopropylate	115.3	110.5	103.7	99.8	93.7	101.7	106.2	104.4	2.0-14.7
Fenpropathrin	115.8	93.3	108.6	91.7	99.6	99.9	102	101.6	9.8- 20.1
Tetradifon	118.6	92.2	93.6	91.1	94.7	108.8	99.2	99.6	8.8-29.6
Phosalone	95.9	107.7	101.8	97.5	95.8	103.2	99.5	100.2	6.3-15.4
Lambda cyhalothrin	102	96.1	92.5	94.51	100.7	103.1	99.2	98.3	3.0- 12.4
Permethrin 1, 2	102.7	115.2	94.5	93.4	94.3	106.4	98.8	188.8	9.2-18.7
Fenvalerate1, 2	105.3	107.9	93.2	95.4	92.3	105.3	99.2	99.8	7.0-25.2
Deltamethrin^a^	.......	.......	.......	96.8	102.4	98.4	100.3	99.4	3.4-15.1

a LOQ = 100 ng/g, n = 12.

**Table 5:** The average and standard deviation of matrix effect on 56 pesticides in rice.

**No.**	**Compound**	**Average matrix effect (%)**	**STDEV** a** (%)**
1	Propoxure 1	77.76	9.8
2	Dichlorvous	86.34	2.6
3	Captan	82.29	0.2
4	Carbaryl	97.66	9.9
5	Propoxure 2	80.05	7.1
6	Diphenyl amine	83.81	7.3
7	Alpha HCH	71.67	0.6
8	Dimethoate	94.42	4.2
9	Gamma HCH	63.34	0.3
10	Beta HCH	63.2	1.3
11	Diazinon	50.65	1.7
12	Etrimfos	68.05	2.3
13	Chlortalonil	76.21	5.1
14	Pirimicarb	79.64	5.4
15	Chlorpyrifos methyl	75.05	6.7
16	Metalaxyl	77.52	14.6
17	Heptachlor	106.03	1.4
18	Alderin	78.09	1.7
19	Fentirothion	80.58	2.1
20	Pirimiphos methyl	74.88	2.8
21	Malathion	93.05	1.7
22	Fenthion	87.62	1.8
23	Chlorpyrifos	83.69	13.9
24	Bioalthrin	87.65	10.7
25	Fipronil	84.47	11.6
26	Heptachlor-exo-epoxide	75.19	12
27	Tridimenol 1	76.64	12.9
28	Heptachlor-endo-epoxide	74.17	13.3
29	Tridimenol 2	82.26	10.6
30	Fenamiphos	136.18	1.5
31	Alpha-Endosulfan	92.51	4.7
32	Oxadiazon	83.31	0.4
33	4,4 DDE	65.85	6.6
34	Dieldrin	80.87	1.1
35	Iprodione	141.57	0.4
36	Beta-Endosulfan	92.12	1.6
37	Ethion	85.62	5.8
38	2,4 DDT	80.68	4.7
39	Propiconazole 1	100.08	0.7
40	Edifenphos	95.98	9.1
41	Propiconazole 2	102.56	5.3
42	4,4 DDT	96.64	5
43	Propargite	96.05	1.2
44	Teboconazole	95.01	8.8
45	Piperonyl botuxide	104.17	0.7
46	Bromopropylate	105.85	10.4
47	Fenpropathrin	112.9	2.3
48	Tetradifon	100.91	5
49	Phosalone	107.8	2.6
50	Lambda cyhalothrin	109.93	9.3
51	Permethrin 1	103.07	3.1
52	Permethrin 2	98.84	0.7
53	Fenvalerate 1	99.34	0.6
54	Fenvalerate 2	136.89	3.3
55	Deltamethrin	79.19	0.5
56	Hexaconazole	108.18	9

a STDEV: Standard deviation.

**Table 6.** Pesticide residues and their concentrations in domestic and imported rice samples in Tehran, Iran.

**Sample**	**Source**	**Pesticide**	**Con. (µg/g)**	**MRL (µg/g)**
1	Domestic	Deltamethrin	1.9± 0.19	0.05
2	Domestic	Deltamethrin	0.19± 0.05	0.05
3	Domestic	Piperonyl botuxide	0.1± 0.04	-----
		Permethrin	0.02± 0.006	2
4	Imported	Carbaryl	0.03± 0.006	1
5	Imported	Diazinon	0.06± 0.01	0.1
		Pirimiphos-methyl	0.03± 0.006	1
6	Imported	Malathion	0.02± 0.008	8
7	Imported	Carbaryl	<LOQ^a^	-----
8	Imported	Piperonyl botuxide	0.01± 0.004	0.05
9	Imported	Gamma HCH	0.01± 0.002	0.05
		Alpha HCH	0.02± 0.003	0.05
10	Domestic	Chlorpryfos	0.02± 0.003	0.1
11	Imported	Chlorpryfos	0.11± 0.02	0.1
12	Domestic	Diazinon	0.01± 0.003	0.1
13	Domestic	Piperonyl botuxide	0.02± 0.005	-----
14	Domestic	Bioalthrin	0.02± 0.004	----

a LOQ = 10 ng/g.

**Table 7.** ADI (μg/kgbw/day) and Estimated Dose (ED) (μg/kgbw/day) for pesticides found in domestic and imported rice samples marketed in Tehran

**Pesticide**	**ADI**	**ED ** ^a^ **, ** ^b^ **, ** ^c^ **, ** ^d^	**ADI (%** e **)**
Carbaryl	8	0.0095	0.095
Diazinon	5	0.0099	0.199
Pirimiphos-methyl	30	0.0077	0.026
Deltamethrin		0.0109	1.09
Permethrin	10	0.0093	0.093
Malathion	300	0.011	0.004
Gamma and Alpha HCH	1	0.009	0.9
Chlorpryfos	10	0.011	0.11
Bioallethrin	------	0.0093	-----
Piperonyl botuxide	200	0.0062	0.003

a : EDs based on mean contamination levels.

b : Using the mean residues. Level < LOQ were considered to be at ½ LOQ.

c : Body weight for adults is assumed 60 kg.

d : Calculated from the mean intake of rice in the Iranian dietetic investigation (110 g) in year 2002-2004.

e : based on mean contamination levels.

The developed method was successfully applied to the analysis of 135 samples of rice collected from Tehran market. A diverse group of pesticide residues such as organophosphorus (diazinon, pirimiphos-methyl, malathion), pyrethroids (deltamethrin, permethrin), carbamate (carbaryl) and benzodioxole (piperonyl botuxide) pesticides were detected in this study. The concentrations of malathion, chlorpryfos, permethrin, diazinon and pirimiphos-methyl were blow the MRLs of these pesticides in Iran. Two rice samples were contaminated with deltamethrin above the MRL. For the other pesticides, no MRL is issued for rice in Iran. In a similar study by Neugen *et al.* in South Korea 6% of the rice samples were contaminated with pesticides (2). They found 88 different pesticides in the samples, and twelve samples were contaminated with more than two pesticides (2).

In a survey conducted by FDA during 1996-2006, ca. 8% of rice samples were found contaminated with pesticides. The most frequently found pesticides included malathion and carbaryl (13). In the present study, fourteen (10.4%) of the 135 samples showed contamination with one of the following pesticides: carbaryl, diazinon, deltamethrin, pirimiphos-methyl, piperonil botuxide, permethrin, bioalthrin, chlorpyrifos and malathion; two samples contained deltamethrin at 0.19 and 1.90 mg/kg; two samples contained carbaryl at <LOQ and 0.03 mg/kg; three samples contained piperonyl botuxide at 0.01, 0.1 and 0.02 mg/kg; one sample contained permethrin at 0.02 mg/kg; two sample contained diazinon at 0.06 and 0.01 mg/kg, one sample contained bioalthrin at 0.02 mg/kg, two samples contained chlorpyrifos at 0.11 and 0.02 mg/kg and one sample contained pirimiphos-methyl at 0.03 mg/kg. Estimated Daily Intake (EDI) of a chemical can be calculated by adding up all the exposures from various pathways. For one contaminant the EDI can be calculated according to the following Equation: 

EDI = EDa + EDw + EDs + EDf + EDws + EDss

The amount of the contaminant as each ED (Estimated Dose) is taken in through a different combination of exposure pathway and the exposure route. In this Equation EDa, EDw, EDs, EDf, EDws, and EDss are the amount inhaled through the air, taken by drinking water, by eating soil, with food, absorbed through skin contact with water and absorbed through skin contact with the soil, respectively (24). In the current study we just calculated the estimated dose of interested pesticides through eating rice and the results demonstrate this pathway has a small portion of ADI.

## Conclusion

A simple and rapid method was developed to determine 56 pesticide residues in rice; a main food in Iranian food basket. The method which consists of a QuEChERS simple sample preparation and GC-SQ-MS-SIM analysis showed a high sensitivity and confirmatory power necessary for the determination of pesticide residues at the levels of maximum residue limits (MRLs) issued in Iran for rice. The excellent method validation data and proficiency test results (Z-score: −0.1 and zero) of the official Food Analysis Performance Assessment Scheme (FAPAS) suggested that the present quantitative method could be applied for rapid determination of pesticides in rice. The developed method has the advantage of using spiked calibration curves that minimizes the matrix interferences leading to higher accuracy for pesticides analyses. Contamination of 5.2% of the analyzed rice samples with unregulated pesticides, (according to Iran’s pesticides regulations) calls for the routine monitoring programs for analysis of pesticide residues in rice. The results show the intakes of eleven detected pesticides found in this study are much lower than the ADIs for them.
